# Supplementary material for: Production of Human IFNγ Protein in Nicotiana benthamiana Plant through an Enhanced Expression System Based on Bamboo mosaic Virus
Source: Viruses. 2019 Jun 3;11(6):509. doi: 10.3390/v11060509 (PMC6630494; doi:10.3390/v11060509)
Supplement: Supplementary file 1 [file viruses-11-00509-s001.pdf]

Supplementary material

Method S1. Details for construction of pKB△C$_{His}$ vector.

To construct pBaMV△CMCS-1, by truncating BaMV CP and inserting the multiple cloning sites (*Cla*I/*Hid*Ш/*Spe*I), a two-step cloning strategy was used as described below. In the first step, the DNA fragment of downstream coding sequence of BaMV TGBp3 was amplified with a plasmid pBaMV as template using specific primer pairs B-8 plus LNK-3 containing *Hid*Ш restriction enzyme site. The amplified PCR fragment was digested with *Ppu10*I and *Hid*Ш and ligated into a pBaMV vector restricted with the same enzyme to generate the recombinant plasmid pBaMV (LINK-3). In the second step, the DNA fragment of coding sequence of BaMV 3′UTR was amplified with a plasmid pBaMV as the template using specific primer pairs 6223SH, containing *Spe*I and *Hid*Ш, plus B21N, containing *Sac*I restriction enzyme sites. The amplified PCR fragment was digested with *Spe*I and *Sac*I and ligated into the pBaMV (LINK-3) vector restricted with the same enzyme to generate the recombinant plasmid pBaMV△CMCS-1, which was further subcloned into a pCass vector to generate pCB△CMCS-1.To construct pCB△CMCS-2 containing putative BaMV CP promoter region, The DNA fragment of the downstream coding sequence of BaMV TGBp3 was amplified with a plasmid pKB as template using specific primer pairs B-8 plus MSNS LIK, containing a series of multiple cloning sites (*Mlu*I/*Stu*I/*Not*I/*Spe*I) and the 5′-terminal 15 nts of BaMV CP coding sequence, of which start codon was mutated. The amplified PCR fragment was digested with *Spe*I and *Nsi*I and ligated into a pCB△CMCS-1 vector restricted with the same enzyme to generate the recombinant plasmid pCB△CMCS-2, which was further subcloned into a pKn binary vector to generate pKB△CMCS-2.

To construct pKB△C$_{His}$ containing 6xHis tag for purification, DNA fragment of BaMV 3′UTR was amplified with a plasmid pkB as the template using specific primer pairs SH-6223, containing coding sequence of six repeats of His and *Spe*I restriction enzyme site, plus B21N, containing the *Sac*I restriction site. The amplified PCR fragment was digested with *Spe*I and *Sac*I, and ligated into a pKB△CMCS-1 vector restricted with the same enzymes to generate the recombinant plasmid pKB△C$_{His}$.

Method S2. Expression of recombinant mIFNγ in *E. coli.*

The coding region of mIFNγ was amplified with the plasmid pKBmIFNγ as a template using specific primer pairs F-*Mlu*I-*Nco*I-mIFNγ, 5′ GCACGCGTCCATGGGCTGTTACTGCC AGGACC-3′ plus R-*Not*I-TGA-His-IFNγ CGGCGGCCGCTCAGTGGTGGTGGTGGTGGTG. The amplified PCR fragment was digested with *Mlu*I and *Not*I and cloned into a plasmid pET28a. The plasmid was then transformed into *E. coli* BL21(DE3) cell for overexpression. Recombinant mIFNγ derived from E. coli was induced by 1 mM IPTG and purified as described the manufacturer’s instructions.

Table S1. List of sense (F) and antisense (R) oligonucleotides used for PCR amplifications.

Primer	Sequence (5' - 3')
F-IFNγ-*Mlu*I	GCACGCGT[1]ATGAAATATACAAGTTATATC
F:*Mlu*I-ATG-IFNγC-1/C-2	GCACGCGT[1]ATGAAGTACACGAGT
F-*Mlu*I-*Nco*I-mIFNγ	GCACGCGTCCATGG[1]GCTGTTACTGCCAGGACC
F:*Mlu*I-*Nco*I-mIFNγC-1	GCACGCGTCCATGG[1]GTTGTTATTGCCAACAT
F:*Mlu*I-*Nco*I-mIFNγC-2	GCACGCGTCCATGG[1]GTTGTTATTgCCAAGAT
R-*Spe*I-IFNγ	CCACTAGT[1]CTGGGATGCTCTTCGACC
R-*Not*I-TGA-His-IFNγ	CGGCGGCCGC[1]TCAGTGGTGGTGGTGGTGGTG
F-P19-*Dra*III	GCC CACGCGGTG[1]ATGGAACGAGCTATACAAGG
R-P19-*Dra*III	GCG CACATGGTG[1]TTACTCGCTTTCTTCTTTGA
F-BaMV4102	CCACTACCAAACAATCAG
R-*Dra*III-p28	GCCACATGGTGT[1]CAAGTGGTCTGGCCAGATG
F-P38 DraIII	GCCCACGCGGTG[1]ATGGAAAATGATCCTAGAGT
R-P38 *Dra*III	GCG CACATGGTG[1] CTAAATTCTGAGTGCTTGCC
R-*Spe*I-SEKDEL-IFNγ	GCACTAGT[1] GAGCTCATCCTTCTCAGA CTGGGATGCTCTTCG
F-SP-*Spe*I	GCACTAGT[1]TCACCCTCTCCAAGCCCTTCCCCATCGCCTAGTCCCTCACCATCC
R-SP-*Spe*I	CGACTAGT[1]TGGGCTGGGAGAAGGGGATGGTGAGGGACTAGGCGA
F-SS-*Mlu*I	GCACGCGT[1]ATGGGGAAAATGGCTTCTCTATTTGCCACTCTTCTAGTAGTTTTAGTGTCA
R-SS-*Mlu*I	CGACGCGT[1]TGCTGAGCTTTCAGAAGCTAAGCTAAGTGACACTAAAACTACTAGAAGAGT
R-BaMV5703	ATCCACTGCTAAGTGTTTGC
F-BCPN	AGGCATCCTATATAATATAC
R-BaMV6366	TGGAAAAAACTGTAGAAACCAAAAGG

[1] Nucleotides underlined indicate restriction enzyme recognition site.

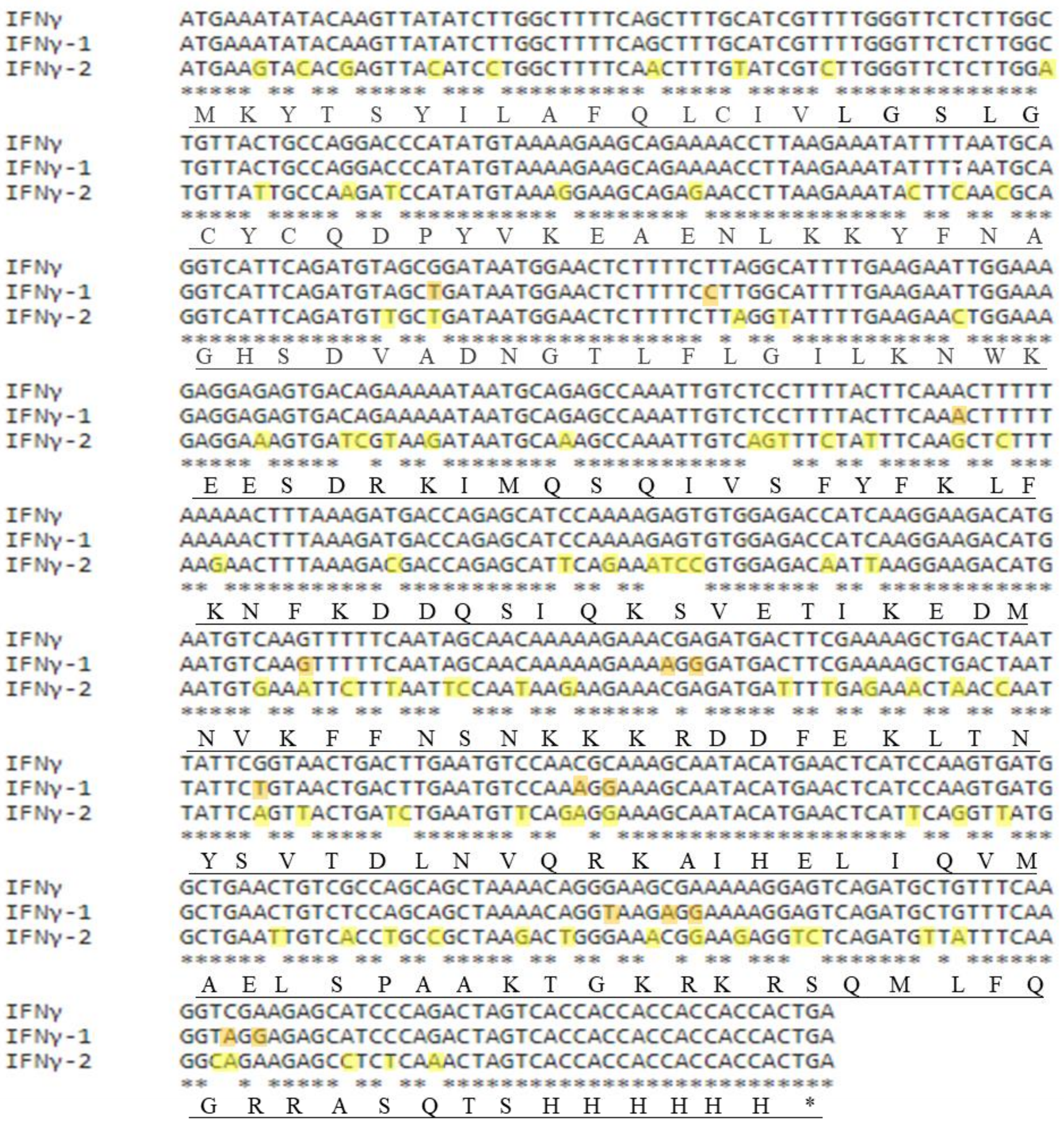

Figure S1. Alignment of codon-optimized nucleotide sequence of IFNγ-1 and -2 with native IFNγ sequence. The codon-optimized IFNγ-1 sequence (14-nucleotide substitutions) was generated based on codon usage table of *N. benthamiana.* Another codon-optimized IFNγ-2 sequence (87-nucleotide substitutions) was generated from the service provided by Integrated DNA technologies (https://sg.idtdna.com/CodonOpt). The codon-optimized IFNγ-1 and IFNγ-2 sequences were aligned to the reference native IFNγ sequence (GenBank accession no. AY121833.1) using Multiple Sequence Alignment. The codon-optimized sites for IFNγ-1 and IFNγ-2 sequences were highlighted by the orange and yellow blocks, respectively.

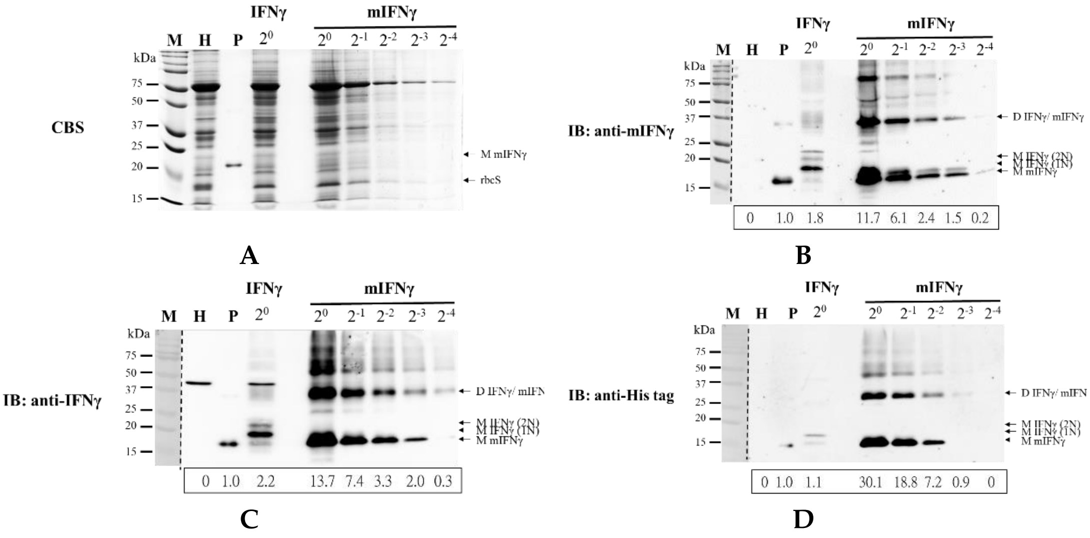

Figure S2. Analysis of preference of different primary antibodies against mIFNγ or IFNγ. *N. benthamina* leaves were infiltrated with A. tumefaciens harboring pKBmIFNγ and pKBIFNγ. Total proteins were analyzed SDS-PAGE, stained by CBS (**A**), and analyzed by immunoblot following the same protocol described in Materials & Methods using different primary antibodies specific to mIFNγ (**B**), N-terminal 1-100 amino acids of native IFNγ (abcam, ab133566) (**C**), and 6X His tag (GeneTex, GTX115045) (**D**). Total protein extracts of IFNγ (2^0) and mIFNγ(2^0) were prepared from infiltrated leaf tissue at 5DPI. In order to verify whether the primary antibodies exhibited preferences against IFNγ or mIFNγ, the mIFNγ protein extract was 2-fold serially diluted. The goat-anti-rabbit IgG horseradish peroxidase conjugate was used as secondary antibody for all immunoblot assays. The signal intensities of mIFNγ or IFNγ in each blot were quantified by densitometry (Image Reader LAS-4000). The numbers below each lane indicate the band intensities of mIFNγ or IFNγ relative to the positive control. M, Marker; H, Healthy leaf; P, Positive control, purified mIFNγ protein derived from E. coli (100 ng for CBS and 10 ng for IB).

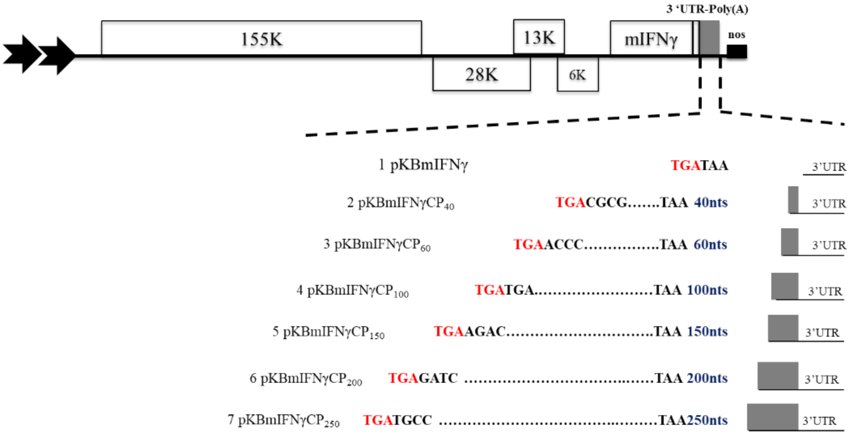
3 'UTR-Poly(A)
155K
13K
mIFNγ
nos
28K
6K
1 pKBmIFNγ TGATAA 3'UTR
2 pKBmIFNγCP40 TGACGCG.......TAA 40nts 3'UTR
3 pKBmIFNγCP60 TGAACCC................TAA 60nts 3'UTR
4 pKBmIFNγCP100 TGATGA.........................TAA 100nts 3'UTR
5 pKBmIFNγCP150 TGAAGAC..............................TAA 150nts 3'UTR
6 pKBmIFNγCP200 TGAGATC ....TAA 200nts 3'UTR
7 pKBmIFNγCP250 TGATGCC ....TAA250nts 3'UTR

A

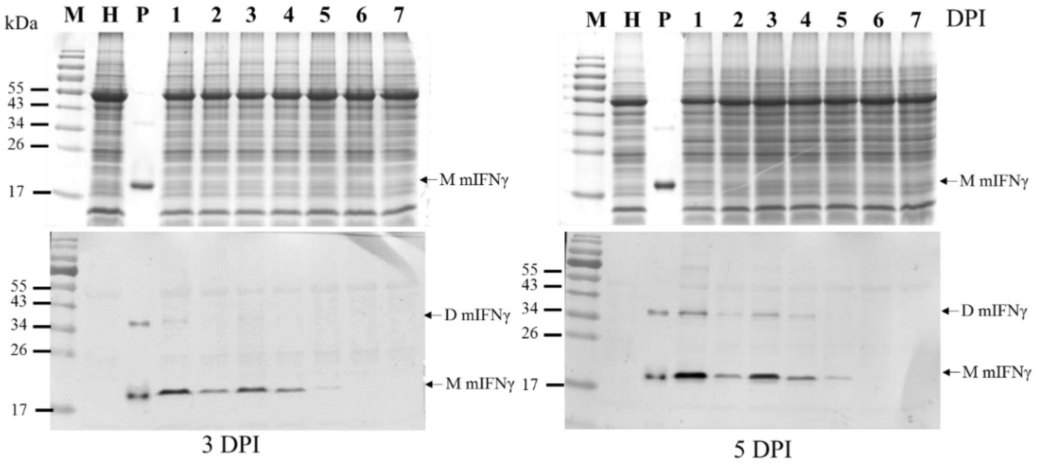
kDa
M H P 1 2 3 4 5 6 7
55
43
34
26
17
M mIFNγ
D mIFNγ
M mIFNγ
3 DPI
M H P 1 2 3 4 5 6 7 DPI
M mIFNγ
D mIFNγ
M mIFNγ
5 DPI

B

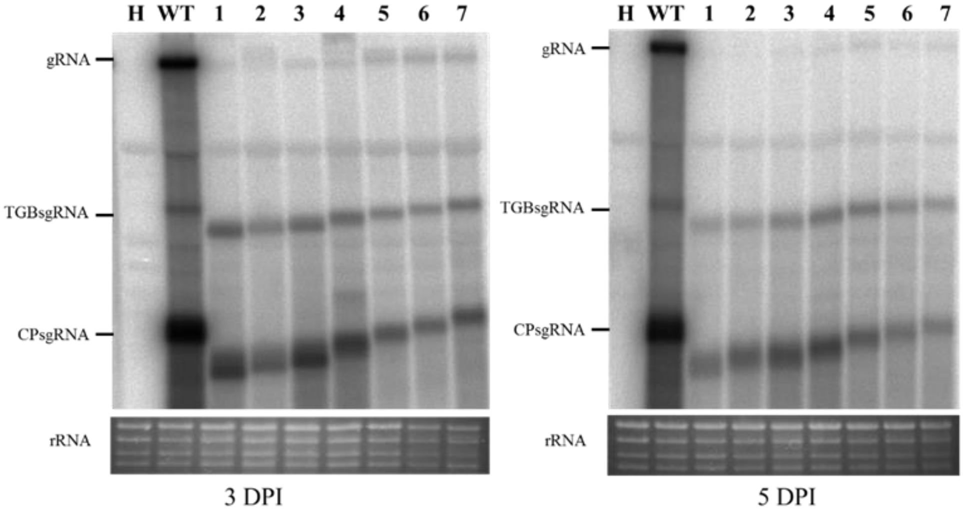
H WT 1 2 3 4 5 6 7
gRNA
TGBsgRNA
CPsgRNA
rRNA
3 DPI
H WT 1 2 3 4 5 6 7
gRNA
TGBsgRNA
CPsgRNA
rRNA
5 DPI

C

Figure S3. The influence of CP C-terminal coding sequence on BaMV replication and IFNγ production. (**A**). Schematic representation of BaMV-based vectors in which various lengths of 3′-terminal nucleotides (positions 40-250) of CP coding region were retained between target protein (TP, mIFNγ) and BaMV 3′ untranslated region (UTR) to generate the following constructs: 1. pKBmIFNγ, 2. pKB△CmIFNγCP40, 3. pKB△CmIFNγCP60, 4. pKB△CmIFNγCP100, 5. pKB△CmIFNγCP150, 6. pKB△CmIFNγCP200, and 7. pKB△CmIFNγCP250. (**B**). Analysis of TP expression in inoculated leaves. Total protein extracts were prepared from infiltrated leaf tissue at 3 and 5 DPI and analyzed by SDS-PAGE, followed by staining with CBS and IB analysis with anti-mIFNγ as primary antibody and goat-anti-rabbit IgG alkaline phosphatase conjugate as secondary antibody. M, Marker; H, Healthy leaf; P, Positive control, purified mIFNγ protein derived from *E. coli* (250 ng for CBS and 25 ng for IB). (**C**). Northern blot analysis of wild-type or chimeric BaMV RNA in infiltrated leaves at 3 and 5 DPI. BaMV genomic RNA, and the subgenomic RNAs for triple gene block proteins (TGPsgRNA) and CP (CPsgRNA) were detected with a BaMV-specific probe (vector, pKB△CHis as control). The bottom panel shows the amount of rRNA in each sample, stained with ethidium bromide as the loading control.
